# Quantification of lung water in heart failure using cardiovascular magnetic resonance imaging

**DOI:** 10.1186/s12968-019-0567-y

**Published:** 2019-09-12

**Authors:** Richard B. Thompson, Kelvin Chow, Joseph J. Pagano, Viktor Sekowski, Evangelos D. Michelakis, Wayne Tymchak, Mark J. Haykowsky, Justin A. Ezekowitz, Gavin Y. Oudit, Jason R. B. Dyck, Padma Kaul, Anamaria Savu, D. Ian Paterson

**Affiliations:** 1 Department of Biomedical Engineering, University of Alberta, Edmonton, Canada; 2 Division of Cardiology, Mazankowski Alberta Heart Institute, University of Alberta, Edmonton, Canada; 3 College of Nursing and Health Innovation, The University of Texas Arlington, Arlington, TX USA; 4 Department of Pediatrics, University of Alberta, Edmonton, Canada; 5 Canadian Vigour Centre, University of Alberta, Edmonton, Canada; 6 University of Alberta Hospital, MacKenzie Health Sciences Centre, 8440–112 street, 2C2.43 Walter C, Edmonton, Alberta T6G2B7 Canada

**Keywords:** MRI, Lung water, Heart failure

## Abstract

**Background:**

Pulmonary edema is a cardinal feature of heart failure but no quantitative tests are available in clinical practice. The goals of this study were to develop a simple cardiovascular magnetic resonance (CMR) approach for lung water quantification, to correlate CMR derived lung water with intra-cardiac pressures and to determine its prognostic significance.

**Methods:**

Lung water density (LWD, %) was measured using a widely available single-shot fast spin-echo acquisition in two study cohorts. *Validation Cohort*: LWD was compared to left ventricular end-diastolic pressure or pulmonary capillary wedge pressure in 19 patients with heart failure undergoing cardiac catheterization. *Prospective Cohort:* LWD was measured in 256 subjects, including 121 with heart failure, 82 at-risk for heart failure and 53 healthy controls. Clinical outcomes were evaluated up to 1 year.

**Results:**

Within the validation cohort, CMR LWD correlated to invasively measured left-sided filling pressures (R = 0.8, *p* < 0.05). In the prospective cohort, mean LWD was 16.6 ± 2.1% in controls, 17.9 ± 3.0% in patients at-risk and 19.3 ± 5.4% in patients with heart failure, *p* < 0.001. In patients with or at-risk for heart failure, LWD >  20.8% (mean + 2 standard deviations of healthy controls) was an independent predictor of death, hospitalization or emergency department visit within 1 year, hazard ratio 2.4 (1.1–5.1, *p* = 0.03).

**Conclusions:**

In patients with heart failure, increased CMR-derived lung water is associated with increased intra-cardiac filling pressures, and predicts 1 year outcomes. LWD could be incorporated in standard CMR scans.

**Electronic supplementary material:**

The online version of this article (10.1186/s12968-019-0567-y) contains supplementary material, which is available to authorized users.

## Background

Pulmonary edema is a central feature of heart failure found in > 50% of patients with acute decompensated [1] or ambulatory disease [2]. It is often characterized by increased intra-cardiac pressure transmitted to the pulmonary vasculature which is a major determinant of fluid accumulation in the pulmonary interstitium and alveolae [3–6]. The resulting pulmonary congestion manifests clinically as dyspnea and is a significant contributor to the severity of exercise intolerance, which is used to stage heart failure and guide treatment [7]. However, these symptoms are not sensitive or specific to heart failure and current imaging tests, such as chest radiography, are qualitative and cannot reliably evaluate the extent of pulmonary fluid accumulation or discriminate non-cardiac causes of dyspnea [8]. Semi-quantitative imaging techniques have been developed to identify pulmonary edema and show promise as a prognostic tool [9, 10].

Cardiovascular magnetic resonance (CMR) is an attractive tool for the assessment of pulmonary edema because the image signal intensity is directly proportional to water density. CMR methods for the quantification of lung water have been validated against gravimetric measurements [11, 12] and used to study regional pulmonary water distribution [13], but have not yet been evaluated in a clinical setting. With the proliferation of CMR for the characterization and management of heart failure [14], a quantitative approach for the assessment of pulmonary edema could provide important complimentary information to clinical scans. The primary goals of this study were to: (1) determine the relationship between CMR-derived lung water content and invasively measured left-sided filling pressure in patients with heart failure (validation cohort) and (2) evaluate its relationship to prognosis in patients with heart failure (prospective cohort).

## Methods

### Validation cohort

The study was approved by the University of Alberta Health Research Ethics Board and written informed consent was given by all study participants. Consecutive patients with a clinical diagnosis of heart failure referred to a tertiary care centre for cardiac catheterization were screened for enrollment. Those aged < 18 years, unable to provide informed consent, or with a contraindication to CMR were excluded. Left ventricular end-diastolic pressure (LVEDP) or pulmonary capillary wedge pressure (PCWP) was measured prior to angiography, with values recorded at end-expiration. Patients underwent CMR within 2 h following the cardiac catheterization once hemostasis had been achieved. B-type natriuretic peptide (BNP) levels were assessed using a Biosite Triage reagent pack (Biosite Inc., San Diego, California, USA) read in an automated Access 2 immunoanalyzer (Beckman-Coulter, Fullerton, California, USA) at Alberta Health Services Laboratory Services- Edmonton, Alberta.

### Prospective cohort

A prospective cohort of healthy controls and patients with or at-risk for heart failure were included from the Alberta HEART study [15]. Patients with heart failure and those at-risk (history of coronary artery disease, diabetes mellitus, hypertension, atrial fibrillation, and/or obesity) were recruited from ambulatory clinics and underwent comprehensive phenotyping that included a detailed history and physical examination, serum biomarkers and a multi-parametric CMR exam. Heart failure patients were sub-grouped into those with preserved left ventricular ejection fractions (HFpEF, LVEF ≥45%) and those with reduced ejection fraction (HFrEF, LVEF < 45%) as prespecified [13]. Age and gender matched controls were also recruited and underwent identical testing.

### MRI protocol

All subjects were imaged on a 1.5 T scanner (Sonata, Siemens Healthineers, Erlangen, Germany). Cardiac structure and function were acquired using balanced steady-state free precession cine sequence with retrospective electrocardiogram (ECG) gating and during 8–12 s breath-holds. Ventricular volumes and mass were measured using commercially available image analysis software, Syngo Argus, (Siemens Healthineers) by an experienced CMR interpreter (IP).

Lung water was measured using a half-Fourier single-shot turbo spin-echo (HASTE) pulse sequence using the body coil for signal excitation and reception. Typical imaging parameters included a matrix size of 128 × 66 with a 360 × 270 mm field of view, 8 mm slice thickness (20 mm gap between slices), 4/8ths partial Fourier, 3.4 ms echo spacing, 780 Hz/pixel bandwidth, 12 ms echo time, a 120°-180° refocusing pulse flip angle and ECG gating with image acquisition during diastasis. Ten to twelve sagittal slices covered the right and left lungs with one image acquired per heartbeat and slice interleaving to minimize T_1_ weighting, with a repetition time > 5 s. Each image acquisition was repeated 3–7 times during free breathing for a total scan time of 5 to 7 min.

An abbreviated protocol was used in the prospective cohort. This included identical imaging parameters but the acquisition of only one sagittal slice in the right lung at the largest cross-sectional area and 20 repeats during free-breathing (repetition time > 5 s) for a total scan time of ~ 2 min. Lung water images were acquired approximately 30 min following supine positioning at the onset of the CMR exam in all subjects.

### Lung water analysis

**Fig. 1:**
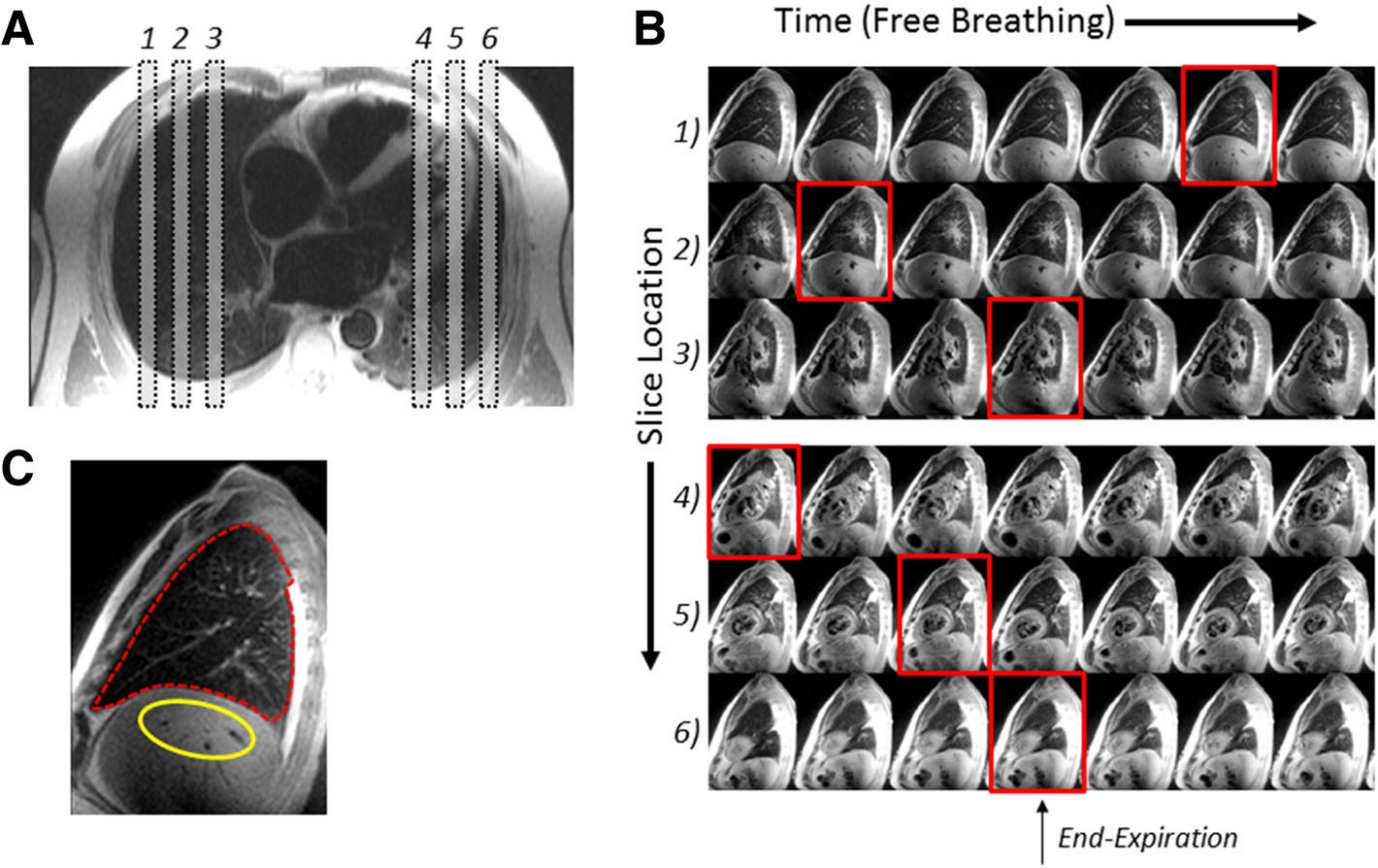
Method for imaging lung water. **a**) Prescription of sagittal slices on a dark blood axial localizer image. Six of 12 slices locations are shown. **b**) Half-Fourier single shot turbo spin echo (HASTE) images acquired during free-breathing at each of the six slice locations with the image closest to end-expiration indicated by a red border. **c**) User-selected regions of interest on the end-expiration images include a tracing of the lung region and a liver region

The lung water acquisition and processing procedure is outlined in Fig. 1. All images were acquired in the sagittal slice orientation (Fig. 1a) during normal tidal respiration with retrospective selection of end-expiration images (Fig. 1b) to minimize lung signal variability due to inflation [16]. Slices with significant contribution from the heart or with small lung volumes in the periphery were discarded. Subsequently, the sagittal cross-section of the lung at end-expiration was manually traced to define the analysis region for each slice, as shown in Fig. 1c, with exclusion of pleural effusion if present. Lung water density (LWD, %) was calculated as the ratio of lung to liver signal intensity multiplied by 70%, the estimated hepatic water density [17].

**Fig. 2:**
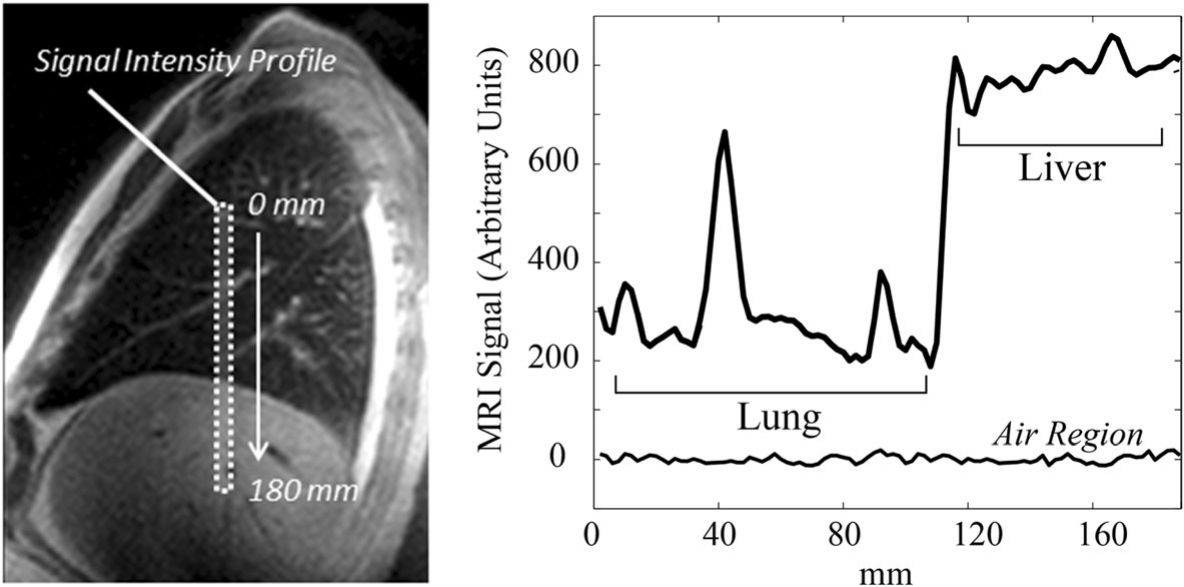
Rectangular profile method for imaging lung water density. The region of interest (10 mm × 180 mm) from which a profile signal intensity is calculated over a central slice in the right lung and liver. A sample signal intensity profile is shown on the right (arbitrary units), showing the relative signal intensities in the lung and liver, and as compared to a noise region, outside of the body

An additional abbreviated 1D lung water analysis was performed in all subjects as shown in Fig. 2. From the sagittal slice of the right lung with the largest cross-sectional area, a thin rectangular region, ~ 10 mm × 150–200 mm, was placed by the interpreter on the centre of the hemidiaphragm to yield a profile of lung and liver signal intensity.

**Fig. 3:**
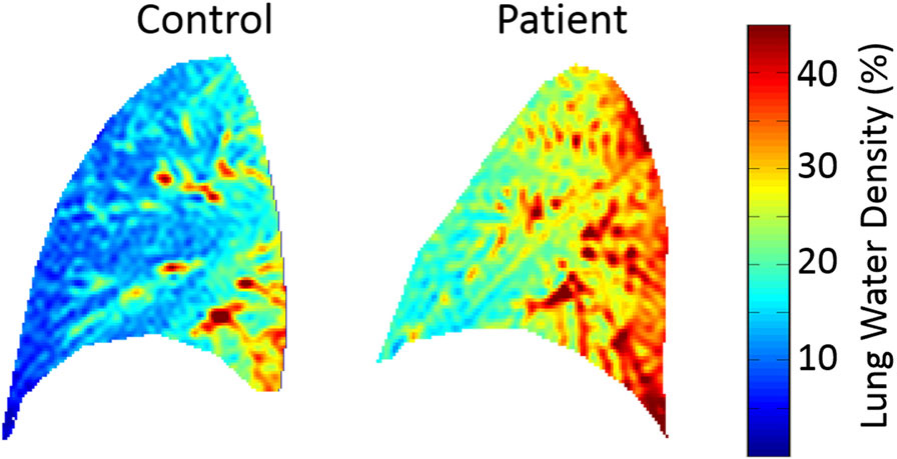
Sample lung water density images. Comparison of lung water density in a healthy control and patient with heart failure after removal of blood vessels and insertion of missing pixels using linear interpolation. Using the rectangular profile analysis method (Fig. 2), the lung water density was 16.5% in the control subject and 27.1% in the patient. The patient had an elevated left ventricular end-diastolic pressure of 31 mmHg (normal ≤12 mmHg) on cardiac catheterization and a brain naturetic peptice (BNP) of 1467 pg/ml (normal < 100 pg/ml)

Lung water density in the validation cohort was derived as the signal average from: (1) the entire lung volume (non-discarded slices), (2) the left or right lung, separately, or (3) the rectangular 1D profile. Figure 3 compares right lung images, in units of water density (%) in a healthy subject and a heart failure patient. LWD in the prospective cohort was measured using the rectangular profile method only. Lung water analyses were performed by two interpreters (VS, RT), blinded to clinical, hemodynamic and serum biomarker data, to evaluate analysis reproducibility in the prospective cohort.

### Statistics and outcomes

Continuous variables are presented as mean ± standard deviation and compared using the Student’s t-test and one-way ANOVA. Categorical variables are presented as counts and frequencies and compared using a χ2-test or exact test. Correlations between lung water density, left-sided filling pressure and BNP were assessed using the Spearman’s rank correlation coefficient. In the prospective cohort, patients with “wet lungs” were identified as LWD greater than the mean + 2 standard deviations for healthy controls (LWD >  20.8%). Clinical outcomes (death, hospitalizations, and emergency department visits) within 1 year from CMR were obtained from in-person follow-up and electronic records. The International Classification of Diseases codes (version 10) recorded in main diagnosis field were used to identify health service utilizations for heart failure or cardiovascular reasons including cerebrovascular bleed/infarction, myocardial ischemic syndrome, arrhythmia and labile blood pressure. Kaplan-Meier curves for time to clinical events within 1 year from CMR were constructed. Proportional hazard regression was used to identify predictors of a composite event (death, hospitalization or emergency department visit for cardiovascular reasons) for patients with or at-risk for heart failure including LWD as well as known predictors of outcomes [18]. The multivariable model with lowest Akaike Information Criterion was identified among models that included three or less significant predictors (*p* < 0.1) from univariate testing. *P* values < 0.05 were considered statistically significant. Coefficient of variation and intra-class correlation coefficient between the two interpreters was calculated for reproducibility analysis. Statistical analysis was generated using MATLAB (R2015a, The MathWorks Inc., Natick, Massachusetts, USA) and SAS software (Version 9.4, SAS Institute, Cary, North Carolina, USA).

## Results

Nineteen patients with heart failure (16 male, age 51 ± 13 years) were recruited into the validation cohort after excluding 4 patients with incomplete hemodynamic data and one with CMR image artifacts. The heart failure etiology included dilated cardiomyopathy in 7, cardiac allograft failure in 4, ischemic cardiomyopathy in 4, valvular heart disease in 3 and pulmonary hypertension in one. Ten of the 19 patients were studied during a hospitalization for acute decompensation while the remaining 9 were tested in an ambulatory setting. Two-hundred and fifty-six subjects (126 male, age 66 ± 11 years), with lung water imaging from the prospective Alberta HEART cohort were included, 121 patients with heart failure, 82 at-risk for heart failure and 53 healthy controls (Table 1).

**Table 1:** Subject Demographics

	Validation Cohort	Prospective Cohort					
		Normal	At-Risk	HFrEF		HFpEF	
				*NYHA I/II*	*NYHA III/IV*	*NYHA I/II*	*NYHA III/IV*
Patients, n		53	82	36	15	52	18
Male sex, n (%)*	19 (84)	16 (30)	44 (54)†	26 (72)†	6 (40)	30 (58)†	4 (22)
Age at CMR, years*	51 ± 13	65 ± 11	64 ± 10	66 ± 10	67 ± 16	68 ± 12	76 ± 9†
Height, cm*	173 ± 11	168 ± 9	170 ± 10	172 ± 10†	166 ± 9	170 ± 9	161 ± 11†
Weight, kg*	89 ± 21	70 ± 12	81 ± 16†	89 ± 19†	85 ± 18†	88 ± 15†	78 ± 19
BMI, kg/m^2*^	29 ± 7	25 ± 3	28 ± 5†	30 ± 5†	31 ± 7†	31 ± 5†	30 ± 8†
HR, min^− 1^	71 ± 22	70 ± 11	68 ± 13	72 ± 17	73 ± 12	69 ± 11	67 ± 12
Systolic BP, mmHg*	137 ± 23	131 ± 21	139 ± 19†	128 ± 21	121 ± 18	129 ± 18	132 ± 17
Diastolic BP, mmHg*	77 ± 15	76 ± 10	82 ± 13†	77 ± 18	71 ± 13	73 ± 14	74 ± 11
Comorbidities, *n*(%)							
Diabetes*	NA	0 (0)	19 (23)†	14 (39)†	7 (47)†	18 (35)†	8 (44)†
Hypertension*	NA	0 (0)	74 (90)†	21 (58)†	8 (53)†	38 (73)†	15 (83)†
CAD/MI*	NA	0 (0)	23 (28)†	22 (61)†	6 (40)†	16 (31)†	7 (39)†
Current smoker*	NA	2 (4)	11 (13)	2 (6)	2 (13)	8 (15)*	0 (0)
COPD*	NA	0 (0)	7 (9)†	5 (14)†	5 (33)†	8 (15)†	7 (39)†
AFib*	NA	0 (0)	16 (20)†	13 (36)†	7 (47)†	24 (46)†	8 (44)†
Medications at baseline, n(%)							
Beta blocker*	NA	0 (0)	32 (39)†	32 (89)†	13 (87)†	43 (83)†	16 (89)†
ACEi or ARB*	NA	0 (0)	64 (78)†	33 (92)†	14 (93)†	41 (79)†	16 (89)†
Laboratory results							
BNP, pg/ml*	505 ± 465	31 ± 20	53 ± 68†	244 ± 256†	381 ± 391†	144 ± 148†	265 ± 205†
Creatinine, umol/L	NA	97 ± 140	82 ± 19	93 ± 25	98 ± 60	105 ± 43	100 ± 36
MRI measurements							
LVEF, %*	37 ± 18	63 ± 6	61 ± 9	32 ± 9†	34 ± 9†	56 ± 8†	59 ± 8†
LVEDVi, ml/m^2*^	122 ± 56	70 ± 11	73 ± 19	119 ± 42†	115 ± 38†	75 ± 24	75 ± 22
LV Massi, g/m^2*^	99 ± 35	52 ± 10	61 ± 14†	86 ± 23†	82 ± 23†	68 ± 13†	65 ± 14†
LV Mass / LVEDV*	0.87 ± 0.25	0.76 ± 0.15	0.85 ± 0.16†	0.75 ± 0.17	0.74 ± 0.19	0.95 ± 0.23†	0.90 ± 0.22†

Note: BNP, and creatinine were missing for 14 subjects. Missing values are not accounted for in reported statistics

Abbreviations *– HFrEF* heart failure with reduced ejection fraction, *HFpEF* heart failure with preserved ejection fraction, *NYHA* New York Heart Association, *CMR* cardiovascular magnetic resonance imaging, *BMI* body mass index, *HR* heart rate, *BP* blood pressure, *CAD/MI* coronary artery disease/myocardial infarction, *COPD* chronic obstructive lung disease, *ACEi* angiotensin converting enzyme inhibitor, *ARB* angiotensin II receptor blocker, *BNP* b-type natriuretic peptide, *LVEF* left ventricular ejection fraction, *LVEDVi* indexed left ventricular end-diastolic volume, *LV massi* indexed left ventricular mass

*p < 0.05 ANOVA comparison across the 6 groups of the Prospective Cohort; †*p* < 0.05 compared to normal controls

### Validation cohort

**Fig. 4:**
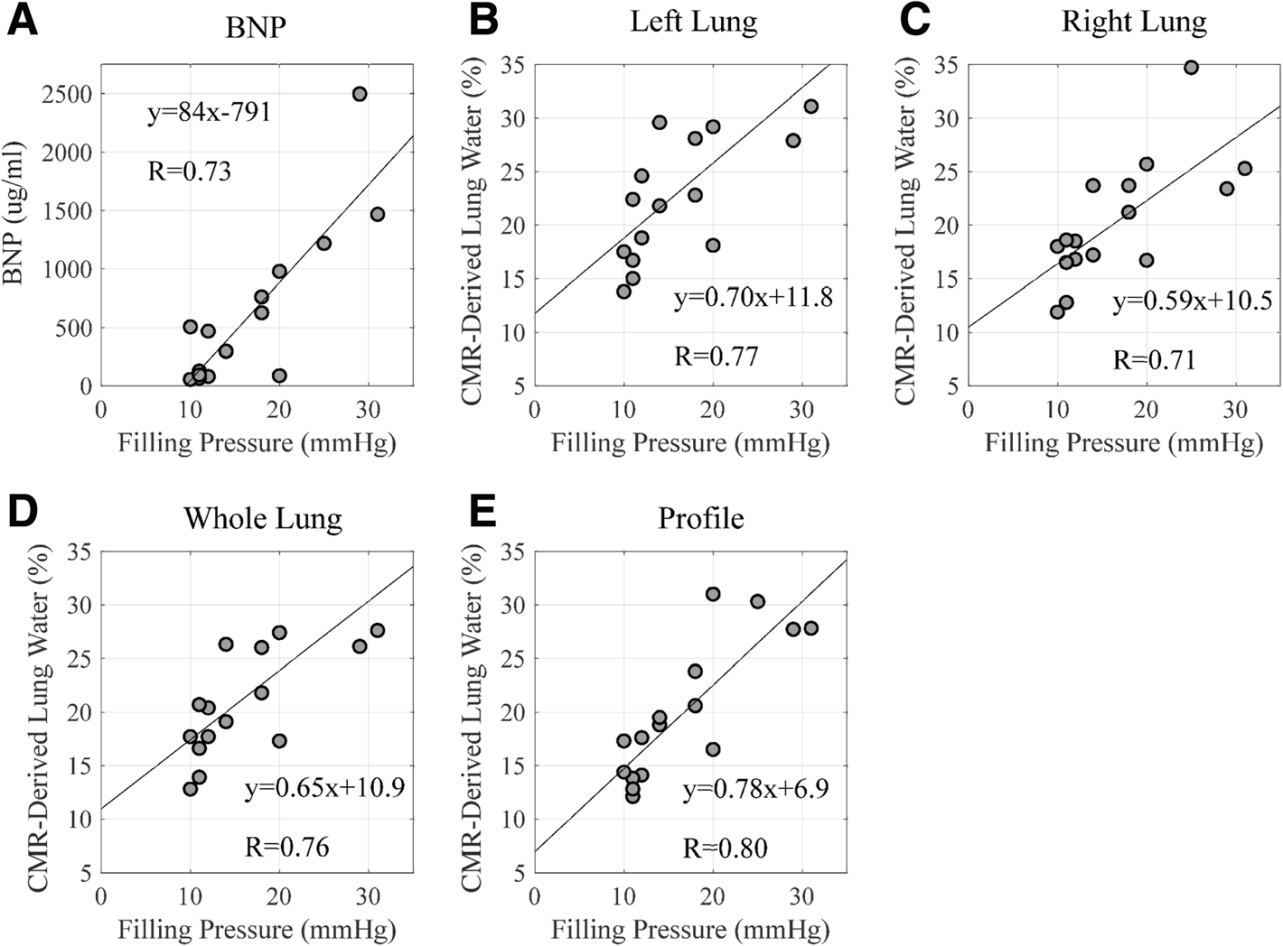
Comparison of lung water density and BNP with filling pressures. Comparison of left sided filling pressures with **a**) BNP, and with CMR derived lung water in the left lung (**b**), the right lung (**c**), the whole lung (**d**) and with the profile method in the right lung (**e**). *p* < 0.05 for each comparison

Mean lung water density was 20.8 ± 5.8% using the rectangular 1D profile method, 23.3 ± 6.5% for the left lung, 19.8 ± 5.5% for the right lung and 21.4 ± 5.8% for whole lung analysis technique. Comparison between CMR post-processing methods (left, right, whole lung and rectangular 1D profile) for LWD yielded R^2^ values ranging 0.88 to 0.98, for all comparisons (*p* < 0.05). The correlation between left-sided filling pressure (LVEDP or PCWP) and LWD ranged from R = 0.71 to 0.80, depending on the image analysis region (Fig. 4). The correlations between BNP and LWD were similar, with R = 0.69 to 0.83 for the different lung water analysis methods. Comparison of left-sided filling pressures and BNP yielded a R = 0.73.

### Prospective cohort

**Fig. 5:**
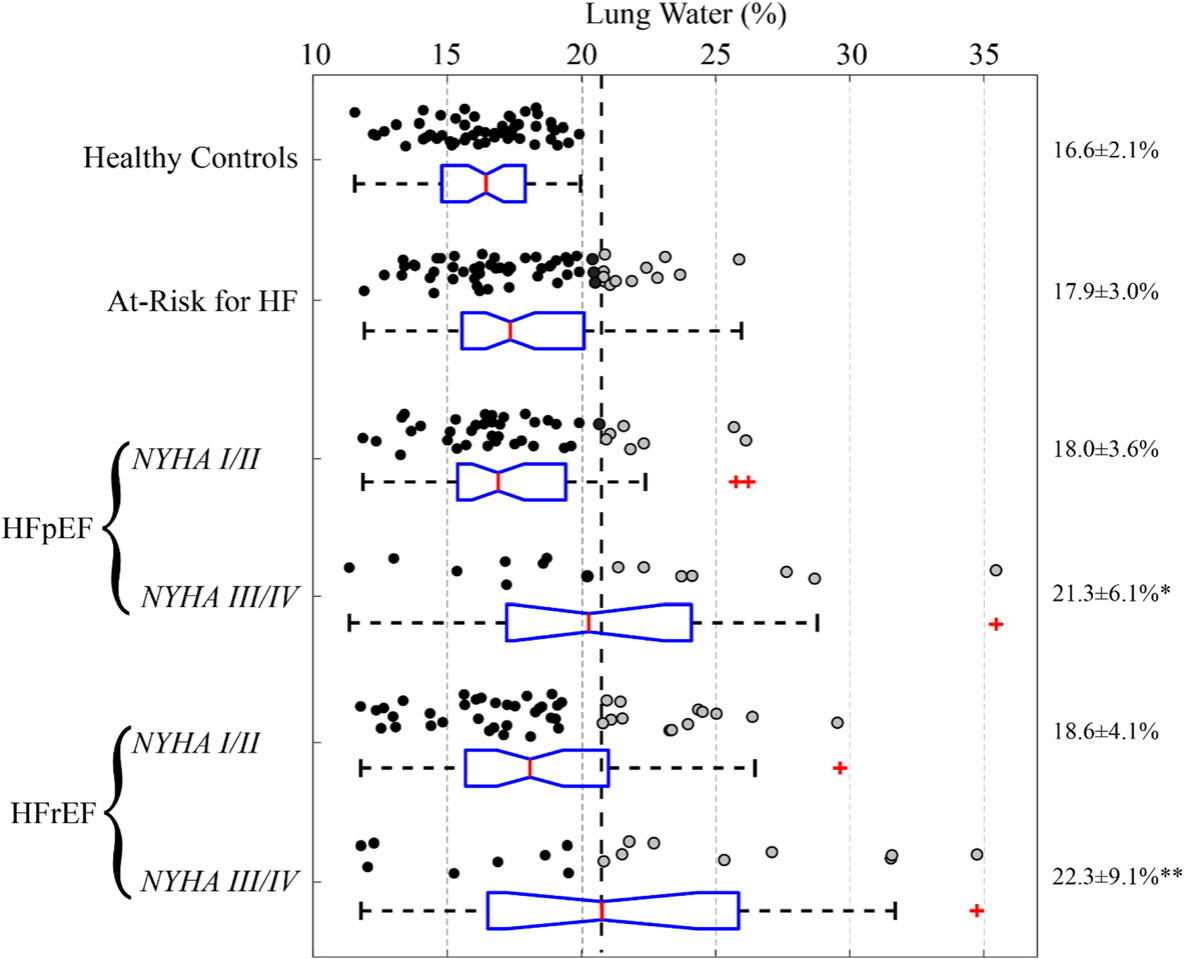
Summary of lung water density using the rectangular profile method in all subjects from the Prospective Cohort. A dashed line, at 20.8%, indicates the upper limit of normal lung water density defined as mean + 2 standard deviations from the Healthy Control group. Each circle is an individual subject with gray denoting individuals above the normal threshold. Box plots for each group show the median, 25th and 75th percentiles and the whiskers show the extent of the data, with red crosses for outliers. Groups with increased lung water, *p < 0.05 in comparison with Healthy Control and At-Risk groups, **p < 0.05 in comparison with Healthy Control, At-Risk and NYHA I/II groups. Abbreviations – HF: heart failure, HFpEF: heart failure with preserved ejection fraction, HFrEF: heart failure with reduced ejection fraction, NYHA: New York Heart Association Classification

Lung water images were evaluated in all 256 subjects from the prospective cohort. Mean LWD was 16.6 ± 2.1% for healthy controls, 17.9 ± 3.0% for patients at-risk for heart failure and 19.3 ± 5.4% for patients with heart failure, *p* < 0.001 for ANOVA (Fig. 5). The proportion of patients exceeding the 20.8% LWD threshold (i.e. wet lungs) defined from the healthy control group was 28% for the at-risk group and 53% in patients with NYHA class III heart failure (HFpEF and HFrEF). No significant difference in lung water was observed between patients with HFrEF and HFpEF, mean LWD 20.0 ± 4.5% vs. 18.7 ± 5.9% respectively, *p* = 0.18. Multiple linear regression analyses demonstrated that lung water density was independent of age and gender.

**Fig. 6:**
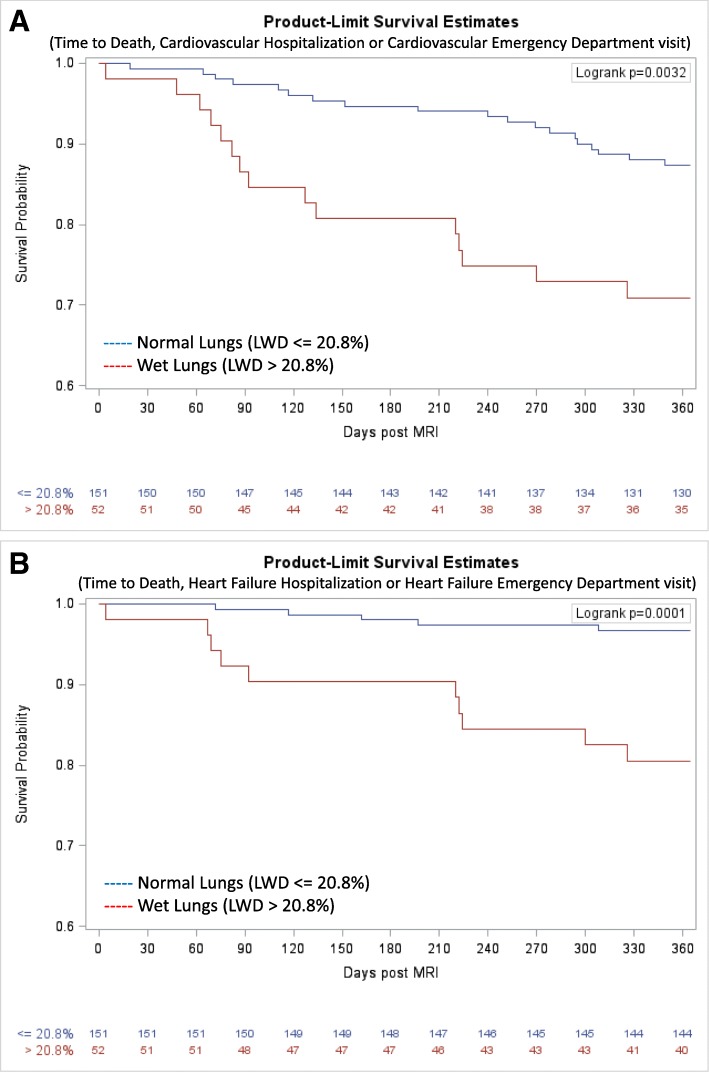
Kaplan-Meier survival curves for 203 patients with or at-risk for heart failure from the Prospective Cohort stratified by lung water density (Panel **a** – Cardiovascular Events, Panel **b** – Heart Failure Events). Abbreviations - LWD: lung water density

Outcome data were available for all subjects in the prospective cohort. At 1 year, death or a cardiovascular event had occurred in 34 participants and was more common in patients with wet lungs (Table 2, Fig. 6). On univariate analysis, proportional hazard regression identified age, history of heart failure, New York Heart Association (NYHA) Class III/IV, beta blocker use, diuretic use, BNP and LWD > 20.8% as having a significant increasing association with the composite outcome, *p* < 0.1 (Table 3). On multivariable analysis, age, BNP and LWD remained significantly associated with outcome. Lung water density > 20.8% predicted death or cardiovascular event in the entire cohort, hazard ratio 2.4 (95% confidence intervals 1.1–5.1, *p* = 0.03) as well as in the subgroup of patients with heart failure, hazard ratio 2.8 (95% confidence intervals 1.2–6.4, *p* = 0.017) (Table 4).

**Table 2:** Clinical Characteristics and Outcomes for the Prospective Cohort, Stratified by Lung Water Density

	CMR derived Lung Water Density Normal (≤ 20.8%) Wet (> 20.8%)		*P* value
Patients, n	151	52	
Male sex, *n*(%)	86 (57)	24 (46)	0.18
Age at CMR, years	67 ± 11	65 ± 12	0.18
BMI, kg/m^2^	29 ± 5	31 ± 7	0.002
Current smoker, *n*(%)	21 (14)	2 (4)	0.048
Diabetes, *n*(%)	48 (32)	18 (35)	0.71
Hypertension, *n*(%)	118 (78)	38 (73)	0.46
CAD/MI, *n*(%)	57 (38)	17 (33)	0.51
Atrial Fibrillation, *n*(%)	51 (34)	17 (33)	0.89
COPD, *n*(%)	20 (13)	12 (23)	0.09
Beta Blocker use, *n*(%)	97 (64)	39 (75)	0.15
ACEi or ARB use, *n*(%)	123 (81)	45 (87)	0.40
Loop diuretic use, *n*(%)	61 (40)	31 (60)	0.016
Spironolactone use, *n*(%)	27 (18)	10 (19)	0.83
History of heart failure	83 (55)	38 (73)	0.022
NYHA, *n*(%)			0.002
Class I	25 (30)	3 (8)	
Class II	43 (52)	17 (45)	
Class III	15 (18)	17 (45)	
Class IV	0 (0)	1 (3)	
Systolic BP, mmHg	133 ± 19	132 ± 22	0.75
Elevated JVP, *n*(%)	25 (17)	19 (37)	0.003
Rales, *n*(%)	5 (3)	7 (13)	0.007
BNP, pg/ml	111 ± 122	282 ± 330	< 0.001
Creatinine, umol/L	91 ± 32	96 ± 39	0.38
LVEF, %, by MRI	54 ± 13	48 ± 19	0.013
Outcomes at 1 year, *n*(%)			
Death	1 (1)	3 (6)	0.022
Death, CV hosp or CV ED visit	19 (13)	15 (29)	0.007

Note: (a) CMR lung water derived by the rectangular profile method; (b) BNP and creatinine were missing for 9 and 5 patients, respectively

Abbreviations – *CMR* cardiovascular magnetic resonance imaging, *BMI* body mass index, *BP* blood pressure, *CAD/MI* coronary artery disease/myocardial infarction, *NYHA* New York Heart Association, *COPD* chronic obstructive lung disease, *ACEi* angiotensin converting enzyme inhibitor, *ARB* angiotensin II receptor blocker, *LVEF* left ventricular ejection fraction, *BNP* b-type natriuretic peptide, *CV* cardiovascular, *hosp* hospitalization, *ED* emergency department

**Table 3:** Univariate Cox Regression Models for Time to 1-Year Composite Outcome in the Prospective Cohort, Stratified by Heart Failure

Univariate Predictors	Patients with or at risk for HF (*N* = 203, 34 events)			Only Patients with HF (*N* = 121, 28 events)		
	HR	95% CI	*p*-value	HR	95% CI	*p*-value
Age at CMR, 10 year	1.9	(1.3, 2.8)	< 0.001	1.8	(1.2,2.6)	0.004
Female gender	1.4	(0.7, 2.8)	0.31	1.8	(0.8, 3.8)	0.13
BMI, 1 kg/m^2^	1.0	(0.9, 1.1)	0.75	0.9	(0.9. 1.0)	0.06
Current smoker	1.0	(0.4, 2.9)	0.96	0.6	(0.2. 2.7)	0.54
Diabetes	1.3	(0.7, 2.6)	0.42	1.2	(0.6. 2.6)	0.62
Hypertension	0.7	(0.3, 1.5)	0.4	0.9	(0.4. 1.9)	0.77
CAD/MI	1.2	(0.6, 2.4)	0.58	0.9	(0.4. 1.8)	0.69
Atrial Fibrillation	1.3	(0.6, 2.6)	0.48	0.9	(0.4. 1.9)	0.73
COPD	1.7	(0.8, 3.8)	0.18	1.3	(0.5, 3.0)	0.58
History of heart failure	3.5	(1.4, 8.4)	0.006	na	na	na
NYHA class III or IV	4.1	(2.0, 8.2)	< 0.001	2.9	(1.4, 6.2)	0.005
Beta blocker at baseline	3.1	(1.2, 8.0)	0.02	1.4	(0.4, 4.5)	0.62
ACEi or ARB at baseline	1.2	(0.5, 3.1)	0.71	0.9	(0.3, 2.8)	0.94
Loop diuretic at baseline	2.4	(1.2, 4.8)	0.02	1.4	(0.6, 3.3)	0.42
Systolic BP, 10 mmHg	1.0	(0.8, 1.1)	0.56	1.1	(0.9. 1.3)	0.56
BNP, 100 pg/ml	1.2	(1.1, 1.3)	< 0.001	1.2	(1.1, 1.3)	< 0.001
Creatinine, 10 umol/L	1	(1.0, 1.2)	0.033	1.0	(0.9, 1.1)	0.53
LVEF, 10%	0.9	(0.7, 1.1)	0.35	1.1	(0.9, 1.5)	0.43
Lung water density > 20.8%	2.6	(1.3, 5.6)	0.005	2.6	(1.3, 5.6)	0.01

Composite outcome: death, cardiovascular hospitalization or cardiovascular emergency department visit

Abbreviations – *HF* heart failure, *HR* hazard ratio, *CI* confidence interval, *CMR* cardiovascular magnetic resonance imaging, *BMI* body mass index, *BP* blood pressure, *NYHA* New York Heart Association, *CAD/MI* coronary artery disease/myocardial infarction, *COPD* chronic obstructive pulmonary disease, *ACEi* angiotensin converting enzyme inhibitor, *ARB* angiotensin II receptor blocker, *BNP* b-type natriuretic peptide, *LVEF* left ventricular ejection fraction

BNP, and creatinine were missing for 9, and 5 patients, respectively. Medians by patient subgroup were used to impute missing values

**Table 4:** Multivariable Cox Regression Models for Time to 1-Year Composite Outcome in the Prospective Cohort, Stratified by Heart Failure

Multivariable Predictors	Patients with or at risk for HF (*N* = 203, 34 events)			Only Patients with HF (*N* = 121, 28 events)		
	HR	95% CI	*p*-value	HR	95% CI	*p*-value
Age at CMR, 10 year	2.2	(1.5, 3.1)	< 0.001	2.2	(1.4, 3.3)	< 0.001
BNP, 100 pg/ml	1.2	(1.1, 1.4)	< 0.001	1.2	(1.0, 1.3)	0.011
Lung water density > 20.8%	2.4	(1.1, 5.1)	0.03	2.8	(1.2, 6.4)	0.017

Composite outcome: death, cardiovascular hospitalization or cardiovascular emergency department visit

Abbreviations – *HF* heart failure, *HR* hazard ratio, *CI* confidence interval, *CMR* cardiovascular magnetic resonance imaging, *BMI* body mass index, *BP* blood pressure, *NYHA* New York Heart Association, *CAD/MI* coronary artery disease/myocardial infarction, *COPD* chronic obstructive pulmonary disease, *ACEi* angiotensin converting enzyme inhibitor, *ARB* angiotensin II receptor blocker, *BNP* b-type natriuretic peptide, *LVEF* left ventricular ejection fraction

BNP, and creatinine were missing for 9, and 5 patients, respectively. Medians by patient subgroup were used to impute missing values

From LWD reproducibility analysis, the intra- and inter-observer coefficient of variation was 3.1 and 3.5%, respectively, and the intra-class correlation coefficient was 0.99.

## Discussion

This is the first reported application of CMR to measure lung water content in a clinical population. We evaluated a simple and widely available free-breathing CMR method for the estimation of lung water density. The major findings were: (1) in a heart failure cohort undergoing cardiac catheterization, CMR-derived LWD correlated to invasively measured left-sided filling pressures; (2) LWD was shown to be less than 20.8% in a healthy control group, with no dependence on age or gender; and (3) in patients with or at-risk for heart failure, LWD > 20.8% predicted clinical outcomes at 1 year in multivariable analysis, with early separation of the survival curves.

### Lung water and filling pressures

While filling pressures were used to confirm an association between LWD and cardiogenic pulmonary edema, a uniform relationship is not expected. Specifically, the accumulation of lung water reflects the integrated effects of capillary wall permeability, hydrostatic and oncotic pressures [19] as well as the rate of active clearance of water from the alveolar space via alveolar epithelial cells [20] and from the interstitial space via the lymphatic system [21]. In cardiogenic pulmonary edema, the main cause of fluid accumulation is increased pulmonary capillary pressure transmitted from the left side of the heart. However, variability in pulmonary fluid accumulation for a given increase in hydrostatic pressure reflects individual adaptations to heart failure. Thus, directly measured intracardiac pressures or commonly used surrogates for filling pressures such as BNP may not be tightly correlated to LWD.

### Existing techniques for evaluating lung water

The need for reproducible and quantitative tests for the evaluation of acute decompensated heart failure, particularly as an entry criteria in therapeutic trials [8], has recently been emphasized, where inconsistent definitions of pulmonary edema are a major limitation [1]. Thermodilution techniques for the measurement of lung water content correlate well to gold-standard gravimetric measures [22], however their clinical use is limited by cost and invasiveness. More recently, the presence of B-lines on lung ultrasonography has been used to diagnose pulmonary edema, however this approach is semi-quantitative and has lower diagnostic accuracy in patients with obesity or co-morbid pulmonary disease [9]. The current study illustrates the feasibility of CMR-derived lung water assessment in a clinical or research setting. In our prospective cohort study of patients with heart failure, diagnostic lung water imaging was obtained in all cases with minimal increase in total scan time. As a standalone test, CMR will not supplant existing tests for pulmonary edema however it may provide important complementary information to CMR examinations increasingly used to manage patients with heart failure [23].

### Lung water density in heart failure

Patients with HFpEF remain a challenging group to manage clinically and drug trials to date have been negative, possibly due to the inclusion of those with non-cardiac causes for exercise intolerance [24]. In fact, several HFpEF phenotypes (e.g. obesity, skeletal muscle weakness, pulmonary hypertension and chronotropic incompetence) with varying degrees of pulmonary congestion have been proposed as a framework for clinical care [25]. In our study, more than half of patients with symptomatic heart failure had normal LWD, irrespective of cardiac function (Additional file 1: Table S1). Similarly, in patients with heart failure and low peak VO2 max on cardiopulmonary exercise testing, we found normal resting lung water in 48% [26]. These findings imply either non-congestive causes of exercise intolerance or exercise induced congestion [27]. Thus in patients with symptomatic HF not responding to medical therapy, lung water imaging has the potential to identify individuals more likely to benefit from non-cardiac interventions [28].

Lung water density independently predicted clinical outcomes for patients with or at-risk for heart failure at 1 year in a multivariable model controlling for known predictors of mortality [18]. Interestingly, we found that LWD and BNP each provided independent information on heart failure prognosis. This finding again supports our earlier assertion that LWD and BNP reflect different pathogenic components of heart failure. Similarly, in a cross-sectional study of 186 patients with heart failure, the quantification of pulmonary congestion on chest x-ray predicted outcome even after adjustment for BNP [10]. Together, these studies suggest that measures of pulmonary congestion are important prognostic and therapeutic targets in heart failure.

### Limitations

There are several approximations in the CMR methodology that could contribute to systematic errors. It was assumed that the liver signal on CMR corresponds to a water density of 70%. Hepatic fatty infiltration or congestion or alternatively iron overload could affect CMR signal intensity and thus lead to an underestimation or overestimation of LWD, respectively [29]. However, water and fat have similar proton densities and thus the replacement of intracellular water by fat should have a minor effect on total received signal. Also, the short echo single-shot spin echo sequence used in the current study is not sensitive to T2* changes, and only weakly to changes in lung or liver T2. In the estimation of lung water density, we assumed an ideal uniformity of the radiofrequency (B_1_) field used for excitation and signal reception. While body coil spatial profiles are considerably more uniform than surface coils, there remains variations in received signal intensities that are independent of water density, particularly at higher static magnetic field strengths. Due to the effects of respiratory phase on lung water signal intensity [30, 31] we only analyzed images at end-expiration. Future work will explore whether breath hold imaging at end expiration is feasible, even in acute heart failure. Another limitation of the current study is the limited spatial coverage provided by the signal profile method which will not reflect regional variations in lung water content. It is possible that water could accumulate non-uniformly and that regional measures, such as the maximum LWD, could be shown to have higher prognostic value. Faster patient-friendly full lung CMR evaluations of water content are needed to address this shortcoming. Also, the redistribution of lung water from upright to supine positioning is unknown. The timing of lung water acquisitions in the prospective cohort were uniform at approximately 30 min into the CMR exam in all subjects. Future studies would ideally characterize these dynamics to allow water redistribution effects over time to be minimized or avoided. While every effort was made to limit the time from cardiac catheterization to CMR in the validation cohort, the approximate 1-h delay between testing could have affected the correlation of measures. Finally, our study is also limited by a single site cohort design that measured LWD predominantly in an outpatient setting at a single time point. Further study of CMR derived LWD in patients with acute decompensated heart failure should be performed to evaluate the effect of diuretic therapy as well as accuracy in those with concurrent lung disease.

## Conclusions

Lung water quantification by CMR is feasible in patients with heart failure. Increased LWD correlates with intra-cardiac filling pressures, is predictive of cardiac events and thus has potential prognostic relevance for patients with heart failure.

## Additional file

Additional file 1: **Table S1.** Clinical Characteristics and Outcomes for Patients with Symptomatic Heart Failure from the Prospective Cohort, Stratified by Lung Water Density. (DOCX 17 kb)

## Data Availability

The datasets used and/or analyzed during the current study are available from the corresponding author on reasonable request.

## Abbreviations

BNP: B-type natriuretic peptide
CMR: Cardiovascular magnetic resonance
ECG: Electrocardiogram
HASTE: Half-Fourier single shot turbo spin echo
HFpEF: Heart failure with preserved ejection fraction
HFrEF: Heart failure with reduced ejection fraction
LVEDP: Left ventricular end-diastolic pressure
LVEF: Left ventricular ejection fraction
LWD: Lung water density
NYHA: New York Heart Association
PCWP: Pulmonary capillary wedge pressure
