# Supplementary material for: Quantification of lung water in heart failure using cardiovascular magnetic resonance imaging
Source: J Cardiovasc Magn Reson. 2019 Sep 12;21:58. doi: 10.1186/s12968-019-0567-y (PMC6739968; doi:10.1186/s12968-019-0567-y)
Supplement: Supplementary file 1 — Table S1. Clinical Characteristics and Outcomes for Patients with Symptomatic Heart Failure from the Prospective Cohort, Stratified by Lung Water Density. (DOCX 17 kb) [file 12968_2019_567_MOESM1_ESM.docx]

**SUPPLEMENTAL TABLE**

Clinical Characteristics and Outcomes for Patients with Symptomatic Heart Failure from the Prospective Cohort, Stratified by Lung Water Density

|  | MRI derived Lung Water Density  Normal ≤ 20.8% Wet > 20.8% | | P value |
| --- | --- | --- | --- |
| Patients, n | 58 | 35 |  |
| Male sex, n(%) | 33 (57) | 16 (46) | 0.3 |
| Age at MRI, years | 72±10 | 67±12 | 0.041 |
| BMI, kg/m^2^ | 30±5 | 32±8 | 0.074 |
| Current smoker, n(%) | 8 (14) | 1 (3) | 0.084 |
| Diabetes, n(%) | 24 (41) | 16 (46) | 0.68 |
| Hypertension, n(%) | 44 (76) | 23 (66) | 0.29 |
| CAD/MI, n(%) | 27 (47) | 14 (40) | 0.54 |
| Atrial Fibrillation, n(%) | 32 (55) | 12 (34) | 0.05 |
| COPD, n(%) | 12 (21) | 11 (31) | 0.24 |
| Beta Blocker use, n(%) | 47 (81) | 31 (89) | 0.34 |
| ACEi or ARB use, n(%) | 45 (78) | 32 (91) | 0.087 |
| Loop diuretic use, n(%) | 44 (76) | 28 (80) | 0.64 |
| Spironolactone use, n(%) | 13 (22) | 10 (29) | 0.51 |
| NYHA, n(%) | | | 0.029 |
| Class II | 43 (52) | 17 (45) |  |
| Class III | 15 (18) | 17 (45) |  |
| Class IV | 0 (0) | 1 (3) |  |
| Systolic BP, mmHg | 128±17 | 128±23 | 0.82 |
| Elevated JVP, n(%) | 22 (38) | 18 (51) | 0.21 |
| Rales, n(%) | 4 (7) | 7 (20) | 0.058 |
| BNP, pg/ml | 183±138 | 355±354 | 0.002 |
| Creatinine, umol/L | 102±43 | 103±45 | 0.97 |
| LVEF, %, by MRI | 48±13 | 45±18 | 0.22 |
| Outcomes at 1 year, n(%) | | |  |
| Death | 1 (2) | 3 (9) | 0.11 |
| Death, HF hosp or  HF ED visit | 4 (7) | 10 (29) | 0.005 |
| Death, CV hosp or  CV ED visit | 10 (17) | 14 (40) | 0.015 |

Note: (a) MRI lung water derived by the rectangular profile method; (b) BNP and creatinine were missing for 4 and 2 patients, respectively.

Abbreviations – MRI: magnetic resonance imaging, BMI: body mass index, BP: blood pressure, CAD/MI: coronary artery disease/myocardial infarction, NYHA: New York Heart Association, COPD: chronic obstructive lung disease, ACEi: angiotensin converting enzyme inhibitor, ARB: angiotensin II receptor blocker, LVEF: left ventricular ejection fraction, BNP: b-type natriuretic peptide, CV: cardiovascular, hosp: hospitalization, ED: emergency department.
